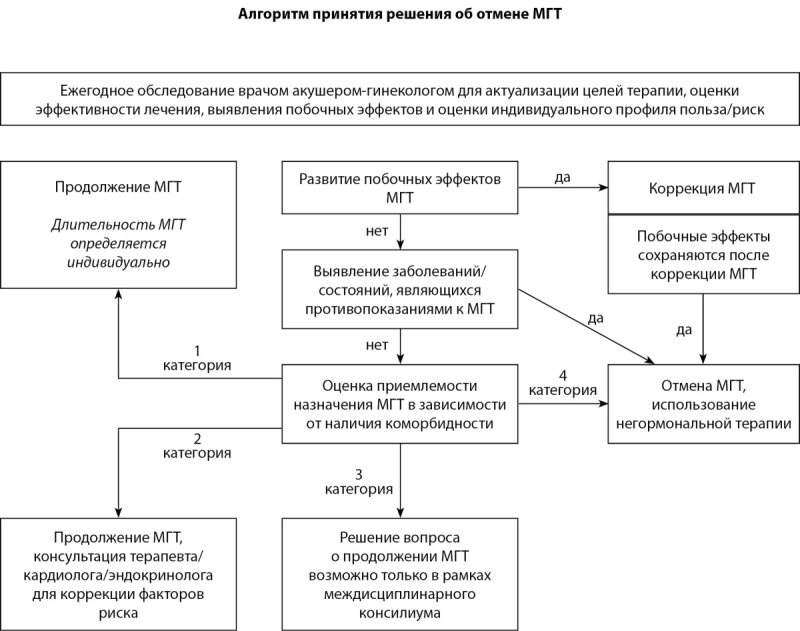# Российские критерии приемлемости назначения менопаузальной гормональной терапии пациенткам с сердечно-сосудистыми и метаболическими заболеваниями. Согласительный документ РКО, РОАГ, РАЭ, ЕАТ, РАФ

**DOI:** 10.14341/probl13394

**Published:** 2023-11-12

**Authors:** Е. В. Шляхто, Г. Т. Сухих, В. Н. Серов, И. И. Дедов, Г. П. Арутюнов, И. А. Сучков, Я. А. Орлова, Е. Н. Андреева, С. В. Юренева, И. С. Явелов, М. И. Ярмолинская, С. В. Виллевальде, О. Р. Григорян, Е. Н. Дудинская, Е. А. Илюхин, Н. А. Козиолова, И. В. Сергиенко, А. А. Сметник, Н. И. Тапильская

**Affiliations:** Национальный медицинский исследовательский центр им. В.А. Алмазова; Национальный медицинский исследовательский центр акушерства, гинекологии и перинатологии им. академика В.И. Кулакова; Национальный медицинский исследовательский центр акушерства, гинекологии и перинатологии им. академика В.И. Кулакова; Национальный медицинский исследовательский центр эндокринологии; Российский национальный исследовательский медицинский университет им. Н.И. Пирогова; Рязанский государственный медицинский университет им. акад. И.П. Павлова; Медицинский научно-образовательный центр МГУ им. М.В. Ломоносова; Национальный медицинский исследовательский центр эндокринологии; Национальный медицинский исследовательский центр акушерства, гинекологии и перинатологии им. академика В.И. Кулакова; Национальный медицинский исследовательский центр терапии и профилактической медицины; Научно-исследовательский институт акушерства и гинекологии и репродуктологии им. Д.О. Отта; Национальный медицинский исследовательский центр им. В.А. Алмазова; Национальный медицинский исследовательский центр эндокринологии; Российский геронтологический научно-клинический центр Российского национального исследовательского медицинского университета им. Н.И. Пирогова; ООО «Медальп»; Пермский государственный медицинский университет им. акад. Е.А. Вагнера; Национальный медицинский исследовательский центр кардиологии им. акад. Е.И. Чазова; Национальный медицинский исследовательский центр акушерства, гинекологии и перинатологии им. академика В.И. Кулакова; Научно-исследовательский институт акушерства и гинекологии и репродуктологии им. Д.О. Отта

**Keywords:** менопаузальная гормональная терапия, сердечно-сосудистые заболевания, метаболические заболевания, сахарный диабет, венозные тромбоэмболические осложнения

## Abstract

Климактерические симптомы могут нарушать ход жизни женщин на пике карьеры и семейной жизни. В настоящее время самым эффективным методом лечения этих проявлений является менопаузальная гормональная терапия (МГТ). Наличие сердечно-сосудистых и метаболических заболеваний само по себе не исключает возможность назначения МГТ с целью купирования климактерических симптомов и улучшения качества жизни. Однако нередко препятствием для использования этого вида гормональной терапии являются опасения врачей, боящихся принести пациенткам больше вреда, чем пользы. Осторожность особенно важна, когда речь идет о женщинах, страдающих сопутствующими заболеваниями. Более того, следует признать, что качественных исследований относительно безопасности МГТ при основных хронических неинфекционных заболеваниях и часто встречаемых коморбидных состояниях недостаточно. В представленном согласительном документе проведен анализ всех доступных в настоящее время данных, полученных в ходе клинических исследований различного дизайна, и создан свод критериев приемлемости назначения МГТ женщинам с сопутствующими сердечно-сосудистыми и метаболическими заболеваниями. Опираясь на представленный документ, врачи различных специальностей, консультирующие женщин в климактерии, получат доступный алгоритм, позволяющий избегать потенциально опасных ситуаций и обоснованно назначать МГТ в реальной практике.

## Abstract (en)

Menopausal symptoms can disrupt the life course of women at the peak of their career and family life. Currently, the most effective treatment for these manifestations is menopausal hormone therapy (MHT). The presence of cardiovascular and metabolic diseases in itself does not exclude the possibility of prescribing MHT to relieve menopausal symptoms and improve quality of life. However, often an obstacle to the use of this type of hormonal therapy is the fear of doctors who are afraid of doing more harm to patients than good. Caution is especially important when it comes to women with underlying health conditions. Moreover, it should be recognized that there is a lack of high-quality research regarding the safety of MHT for major chronic non-infectious diseases and common comorbid conditions. The presented consensus document analyzed all currently available data obtained from clinical trials of various designs and created a set of criteria for the acceptability of prescribing MHT to women with concomitant cardiovascular and metabolic diseases. Based on the presented document, doctors of various specialties who advise women in menopause will receive an accessible algorithm that will allow them to avoid potentially dangerous situations and reasonably prescribe MHT in real practice.

Сопредседатели: Е.В. Шляхто, Г.Т. Сухих, В.Н. Серов, И.И. Дедов, Г.П. Арутюнов, И.А. Сучков

Ответственный секретарь рабочей группы: Я.А. Орлова

Рабочая группа: Е.Н. Андреева, С.В. Юренева, И.С. Явелов, М.И. Ярмолинская, С.В. Виллевальде, О.Р. Григорян, Е.Н. Дудинская, Е.А. Илюхин, Н.А. Козиолова, И.В. Сергиенко, А.А. Сметник, Н.И. Тапильская

Эксперты: Н.В. Артымук, А.Г. Арутюнов, В.Е. Балан, И.И. Баранов, С.А. Бобров, Р.И. Габидуллина, Н.Ю. Григорьева, И.В. Губарева, О.В. Дженина, Ю.Э. Доброхотова, С.О. Дубровина, Е.В. Енькова, Е.И. Ермакова, С.К. Зырянов, Н.Ю. Каткова, Л.Ю. Карахалис, Т.В. Кирсанова, Т.Ю. Кузнецова, Т.А. Макаренко, Л.И. Мальцева, С.В. Мальчикова, С.В. Недогода, С.Ю. Никулина, Т.А. Обоскалова, М.М. Петрова, А.Г. Плисюк, В.И. Подзолков, Н.М. Подзолкова, А.Э. Протасова, И.В. Савельева, Е.А. Сандакова, И.В. Сахаутдинова, М.С. Селихова, Т.М. Соколова, Л.С. Сотникова, Н.В. Спиридонова, Е.И. Тарловская, И.В. Фомин, М.Б. Хамошина, А.И. Чесникова, Г.А. Чумакова, И.И. Шапошник

## СПИСОК СОКРАЩЕНИЙ

## ВВЕДЕНИЕ

Распоряжением Правительства РФ от 29.12.2022 г. № 4356-р утверждена Национальная стратегия действий в интересах женщин на 2023–2030 гг. Одной из важных задач государственной политики становится сохранение здоровья женщин всех возрастов, улучшение качества жизни и увеличение периода активного долголетия [1]. Для реализации этой стратегии в здравоохранении крайне важен междисциплинарный подход. Врачам-интернистам совместно с врачами акушерами-гинекологами необходимо выявлять женщин, вступивших в период менопаузального перехода, для своевременного оказания им необходимой помощи.

Климактерические симптомы могут нарушать ход жизни женщин на пике карьеры и семейной жизни: 75% женщин 45–55 лет предъявляют жалобы на приливы; в 28,5% случаев это приливы средней или тяжелой степени выраженности; продолжительность симптомов может составлять 3–15 лет [2]. В настоящее время самым эффективным методом лечения этих проявлений является МГТ [3][4].

Наличие сердечно-сосудистых и метаболических заболеваний само по себе не исключает возможность назначения МГТ с целью купирования климактерических симптомов и улучшения качества жизни. Однако нередко препятствием для использования этого вида гормональной терапии являются опасения врачей, боящихся принести пациенткам больше вреда, чем пользы.

Осторожность особенно важна, когда речь идет о женщинах, страдающих сопутствующими заболеваниями. Более того, следует признать, что качественных исследований относительно безопасности МГТ при основных хронических неинфекционных заболеваниях и часто встречаемых коморбидных состояниях недостаточно.

Таким образом, цель согласительного документа:

провести анализ всех доступных в настоящее время данных, полученных в ходе клинических исследований различного дизайна, и создать свод критериев приемлемости назначения МГТ женщинам с сопутствующими сердечно-сосудистыми и метаболическими заболеваниями.

Опираясь на представленный документ, врачи различных специальностей, консультирующие женщин в климактерии, получат доступный алгоритм, позволяющий избегать потенциально опасных ситуаций и обоснованно назначать МГТ в реальной практике.

## РАЗДЕЛ 1. ОСНОВНЫЕ ОПРЕДЕЛЕНИЯ, СИМПТОМЫ И КЛАССИФИКАЦИЯ МЕНОПАУЗЫ

Менструальный цикл является одним из важнейших показателей здоровья женщины, и его регулярность может меняться в зависимости от стадии репродуктивного старения.

Рабочая Группа по изучению стадий старения репродуктивной системы женщин (Stages of Reproductive Aging Workshop — STRAW) [5] выделяет три стадии репродуктивного старения: репродуктивная стадия, менопаузальный переход и постменопауза. Классификация этапов старения репродуктивной системы женщин STRAW+10 представлена на рисунке 1.1.

**Figure:**
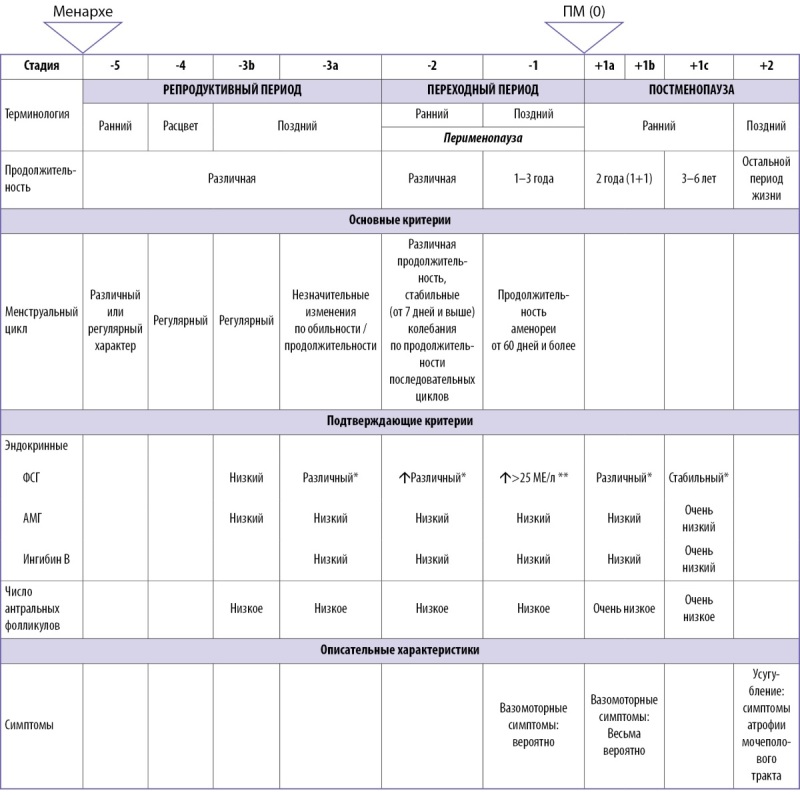
Рисунок 1.1. Классификация этапов старения репродуктивной системы женщин (STRAW+10).При ПНЯ, СПЯ, после гистерэктомии, на фоне приема КОК, ВМС-ЛНГ- критерии STRAW+10 неприменимы.

Менопаузальный переход характеризуется нарушением регулярности менструальных циклов, являющимся отражением вариабельности гормональной секреции и овуляторной функции.

Менопауза — стойкое прекращение менструаций, это последняя самостоятельная менструация, обусловленная возрастным снижением гормональной активности и «выключением» репродуктивной функции яичников. Дата наступления менопаузы оценивается ретроспективно: спустя 12 мес. отсутствия менструации [6][7].

Перименопауза включает период менопаузального перехода + 1 год после последней менструации.

Перименопауза начинается с нарушения регулярности менструального цикла («фаза менопаузального перехода») и длится до 1 года после полного прекращения менструаций. Эта фаза репродуктивного старения может наступать в широком возрастном диапазоне (от 42 до 58 лет) и длиться до 4–8 лет [8].

Постменопауза — период жизни после последней менструации.

Климактерический синдром — комплекс вегетативно-сосудистых, психических и обменно-эндокринных нарушений, возникающих у женщин на фоне угасания (или резкой потери) гормональной функции яичников и общего старения организма [9].

Средний возраст наступления менопаузы во всем мире составляет 48,8 года (95% ДИ 48,3–49,2) со значительными колебаниями этого показателя в зависимости от географического региона проживания женщин [10], в РФ он находится в диапазоне от 49 до 51 года [9]. Распространенность климактерических симптомов вариативна и зависит от ряда обстоятельств.

Вазомоторные симптомы чаще возникают в позднем периоде менопаузального перехода и особенно выражены в перименопаузе и первые годы постменопаузы [11][12]. Вазомоторными симптомами страдают до 80% женщин в перименопаузе [13]. Нарушения сна встречаются 39–47% у женщин в перименопаузе и в 35–60% в постменопаузе [14]. Среди лиц в возрасте 50 лет и старше в РФ остеопороз выявляется у 34% женщин, а частота остеопении составляет 43% [15].

Вазомоторные симптомы и другие проявления климактерического синдрома не только ухудшают качество жизни женщин и ограничивают их функциональные возможности, но и ассоциированы с повышением риска развития ИБС в 1,34 раза, риска любых ССЗ — в 1,48 раза [16].

У 15% женщин в перименопаузе и до 80% женщин в постменопаузе отмечаются симптомы генитоуринарного менопаузального синдрома (ГУМС) или вульвовагинальной атрофии (ВВА) [17]. У 41% женщин в возрасте 50–79 лет есть хотя бы один из симптомов ВВА. Распространенность нарушений мочеиспускания (внезапное и непреодолимое желание помочиться, которое невозможно отсрочить, недержание мочи) у женщин зависит от длительности постменопаузы и увеличивается с 15.5% при постменопаузе до 5 лет и до 41,4% при длительности постменопаузы более 20 лет [17].

Классификация менопаузы

По времени наступления выделяют:

По причине наступления выделяют естественную и ятрогенную (в том числе хирургическую менопаузу).

## РАЗДЕЛ 2. ПОКАЗАНИЯ И ПРОТИВОПОКАЗАНИЯ К МГТ

Показания и противопоказания к назначению МГТ определяются актуальными Клиническими рекомендациями и инструкциями к конкретным препаратам.

Показания к назначению МГТ [4].

Противопоказания к назначению МГТ [4].

## РАЗДЕЛ 3. ВИДЫ МГТ И ОСНОВНЫЕ ПРИНЦИПЫ ЕЕ НАЗНАЧЕНИЯ

Системная МГТ

Системная МГТ является наиболее эффективным методом лечения вазомоторных симптомов и других климактерических проявлений, включая ГУМС. Большинство лекарственных препаратов МГТ одобрены для профилактики постменопаузального остеопороза, за исключением ультранизкодозированных форм.

В таблице 3.1 представлены зарегистрированные на территории РФ препараты для системной МГТ.

**Table:** Таблица 3.1. Зарегистрированные в РФ лекарственные препараты и их комбинации для системной МГТ

Комбинированная терапия эстроген/гестаген в циклическом режиме(в перименопаузе)
Фиксированные комбинации (эстроген/гестаген)
Эстрадиол/Дидрогестерон (1 мг/10 мг; 2 мг/10 мг)
Эстрадиола валерат (2 мг)/Левоноргестрел (150 мкг)
Эстрадиола валерат (2 мг)/Норгестрел (500 мкг)
Эстрадиола валерат (2 мг)/Ципротерона ацетат (1 мг)
Свободные комбинации 2 препаратов (эстроген/гестаген)
Эстрадиола валерат 2 мг	Микронизированный прогестерон 200 мг
Дидрогестерон 10 мг
Эстрадиола гемигидрат гель трансдермальный 0,6 мг/г	Микронизированный прогестерон 200–400 мг Дидрогестерон 10–20 мг
Эстрадиола гемигидрат гель трансдермальный 0,1% — 0,5 г; 1,0 г; 1,5 г	Дидрогестерон 10 мг
Монофазная комбинированная терапия эстроген/гестаген в непрерывном режиме(в постменопаузе)
Фиксированные комбинации
Эстрадиол/Дидрогестерон (0,5 мг/2,5 мг; 1 мг/5 мг)
Эстрадиол/Дроспиренон (0,5 мг/0,25 мг, 1 мг/2 мг)
Свободные комбинации 2 препаратов (эстроген/гестаген)
Эстрадиола валерат 2 мг	Внутриматочная система, содержащая 52 мг левоноргестрела микронизированного (ЛНГ-ВМС)
Эстрадиола гемигидрат гель трансдермальный 0,6 мг/г	Микронизированный прогестерон (100–200 мг)
Внутриматочная система, содержащая 52 мг левоноргестрела микронизированного (ЛНГ-ВМС)
Прогестерон гель вагинальный 8% 90 мг/доза
Эстрадиола гемигидрат гель трансдермальный 0,1% — 0,5 г; 1,0 г; 1,5 г	Микронизированный прогестерон (100–200 мг)
Внутриматочная система, содержащая 52 мг левоноргестрела микронизированного (ЛНГ-ВМС)
Прогестерон гель вагинальный 8% 90 мг/доза
Прочие эстрогены
Тиболон 2,5 мг
Монотерапия эстрогенами(для женщин после гистерэктомии)
Эстрадиола валерат 2 мг
Эстрадиола гемигидрат гель трансдермальный 0,6 мг/г
Эстрадиола гемигидрат гель трансдермальный 0,1% — 0,5 г; 1,0 г; 1,5 г

Локальная МГТ

Локальная терапия эстрогенами (эстриолом) используется у женщин пери- и постменопаузального периода с жалобами только на симптомы ГУМС: сухость влагалища, диспареунию или дискомфорт при половой жизни, связанные с этим состоянием.

Длительные наблюдения (6–24 мес) показывают отсутствие влияния локальных эстрогенов на эндометрий, поэтому не требуется дополнительного использования прогестагенов. Локальные эстрогены не повышают риск ВТЭО, РМЖ, ССЗ, гиперплазии и рака эндометрия по данным наблюдательных исследований [18]. В таблице 3.2 представлены зарегистрированные на территории РФ препараты для локальной МГТ.

**Table:** Таблица 3.2. Зарегистрированные в РФ лекарственные препараты для локальной МГТ

Эстриол (крем вагинальный 1 мг/г, суппозитории вагинальные 0,5 мг)
Эстриол микронизированный 0,2 мг / прогестерон микронизированный 2 мг/лактобактерии (капсулы вагинальные)
Эстриол 50 мкг/г (гель вагинальный)
Эстриол 0,03 мг/лактобактерии (таблетки вагинальные)

Основные принципы назначения МГТ

## РАЗДЕЛ 4. МГТ У ПАЦИЕНТОК С ОЖИРЕНИЕМ И НАРУШЕНИЯМИ УГЛЕВОДНОГО ОБМЕНА

Инсулинорезистентность, дислипидемия, артериальная гипертензия и абдоминальное ожирение — основные маркеры менопаузального метаболического синдрома [19]. По сравнению с репродуктивным периодом, женщины в период перименопаузы и ранней постменопаузы подвержены более высокому риску прогрессирования инсулинорезистентности [20]. С возрастом риск развития метаболического синдрома (МС) увеличивается у женщин в 5 раз. Частота ССЗ повышается у женщин с нарушениями углеводного обмена в 5 раз [21].

Ожирение, особенно абдоминальное, тесно ассоциировано с МС, значительно повышает кардиометаболический риск и отражается на заболеваемости, прогнозе и продолжительности жизни больных [22].

Ожирение является независимым фактором риска развития ВТЭО. В рандомизированном исследовании «Инициатива во имя здоровья женщин» (WHI) у женщин с ожирением (ИМТ >30 кг/м²) было отмечено 3-кратное увеличение риска ВТЭО по сравнении с женщинами с нормальным ИМТ даже в группе плацебо [23].

При ожирении нежелательно назначать препараты, содержащие гестагены с остаточной андрогенной и глюкокортикоидной активностью, предпочтение отдается метаболически нейтральным прогестагенам [24]. После обнаружения связи минералокортикоидных рецепторов с дифференциацией жировой ткани установлена потенциальная роль прогестерона и прогестинов с антиминералокортикоидными свойствами в контроле массы тела и пролиферации жировой ткани [25]. По данным сравнительного исследования назначения комбинированной МГТ, содержащей дроспиренон или дидрогестерон, у пациенток с менопаузальным метаболическим синдромом было показано достоверное снижение веса через 6 мес терапии (c 74,2 до 72,4 кг в группе Э/ДДГ (p=0,03) и с 74,5 до 72,7 кг в группе Э/ДРСП (p=0,05)). Было отмечено улучшение показателей уровня глюкозы натощак (p<0,05) в обеих группах, улучшение показателей HOMA-IR (p=0,03) и MAGE было отмечено в группе Э/ДРСП (p<0,001) [26].

Частота СД 2 типа в популяции женщин составляет: в 40–44 года — 1,2%, в 45–49 лет — 2,4%, в 50–54 года — 4,2%,в 55–59 лет — 9,4% [27]. Своевременное начало МГТ может отложить риск развития СД 2 типа. По данным WHI, терапия КЭЭ + MПA статистически значимо снижала заболеваемость СД 2 типа — на 19% (ОР 0,81; 95% ДИ 0,70–0,94; P=0,005), что соответствует снижению на 16 случаев в пересчете на 10 000 женщин-лет. В когорте монотерапии КЭЭ число новых диагнозов СД 2 типа сократилось на 14% (ОР 0,86; 95% ДИ 0,76–0,98), что соответствует снижению на 21 случай в пересчете на 10 000 женщин-лет [28].

По данным метаанализа 107 исследований, МГТ снижает риск развития СД 2 типа на 30% (ОР=0,7; ДИ 95%=0,6–0,9), а при уже имеющемся СД на фоне МГТ происходит снижение уровня глюкозы натощак и HOMA-IR, а также наблюдается улучшение липидного профиля и снижение АД, наряду со снижением степени абдоминального ожирения. На фоне монотерапии эстрогенами или комбинированной МГТ у женщин с СД2 типа не было отмечено увеличение риска сердечно-сосудистой смертности [29].

При СД 2 типа предпочтителен пероральный вид МГТ, при отсутствии противопоказаний. При назначении комбинированной МГТ важно учитывать метаболические эффекты гестагена, входящего в состав комбинированной МГТ: следует остановить выбор на прогестагенах с нейтральным воздействием на метаболические процессы [30].

Благоприятный эффект МГТ на углеводный обмен прекращается при отмене терапии.

Таким образом, МГТ может быть рассмотрена в качестве терапией менопаузальных симптомов у пациенток с СД 2 типа.

Совместимость сахароснижающей терапии с МГТ, заместительной терапии левотироксином натрия (L-T4), тиреостатической и дофаминергической терапией с учетом путей введения отражена в табл. 4.1 [31].

**Table:** Таблица 4.1. Совместимость МГТ и других фармакологических групп в эндокринологии Примечание: цифра 1 — прием данной терапии на фоне МГТ безопасен, противопоказаний не имеет. Цифра 2 — прием данной терапии на фоне МГТ в целом безопасен, может потребоваться титрация одного/двух компонентов.*При инициации терапии L-T4 может потребоваться коррекция его дозировки во избежание фибрилляции предсердий и остеопороза.**Прием МГТ не влияет на размер микро/макропролактиномы.

Группа препаратов	Комбинированная МГТ	Только эстроген-содержащая МГТ	Тиболон	Локальная МГТ
ПО Э/Г	ТД Э/Г	ПО Э	ТД Э
Пероральная сахароснижающая терапия	1	1	1	1	1	1
Инсулинотерапия	1	1	1	1	1	1
L-T4*	1	1	1	1	1	1
Тиреостатики	1	1	1	1	1	1
Агонисты дофамина**	2	2	2	2	2	1

Ключевые положения

## РАЗДЕЛ 5. МГТ У ПАЦИЕНТОК С ТРОМБОФИЛИЯМИ, ЗАБОЛЕВАНИЯМИ ВЕН, ВЕНОЗНЫМИ ЭМБОЛИЯМИ

## 5.1. Состав МГТ и риск венозных тромбоэмболических осложнений

Считается, что МГТ с использованием в ее составе пероральных эстрогенов повышает риск венозных тромбоэмболических осложнений (ВТЭО) — тромбоза глубоких вен (ТГВ) и тромбоэмболии легочных артерий (ТЭЛА) [32][33]. Однако этот эффект, отмеченный в рандомизированных контролируемых исследованиях и выполненных на их основе метаанализах, может быть во многом связан с назначением достаточно «тромбогенных» препаратов на основе КЭЭ и МПА, а также с несвоевременным началом МГТ.

Так, по данным анализа крупных баз данных QResearch и CPRD, выполненного с использованием метода «случай-контроль», назначение комбинированной МГТ КЭЭ в сочетании с МПА ассоциировалось с наиболее высоким риском ВТЭО. Для перорального эстрадиола было отмечено достоверное повышение риска ВТЭО, и этот эффект был дозозависимым. В то же время для комбинации перорального эстрадиола с дидрогестероном риск ВТЭО не увеличивался ни при циклическом, ни при монофазном комбинированном режимах МГТ вне зависимости от дозы эстрадиола. Назначение трансдермального эстрадиола не было связано с увеличением риска ВТЭО как при монотерапии, так и в составе комбинированной МГТ. Вне зависимости от ИМТ, назначение комбинации перорального эстрадиола с дидрогестероном, трансдермального эстрадиола как в монотерапии, так и в комбинации с гестагеном не было связано с увеличением риска ВТЭО. В когорте женщин, имевших в анамнезе эпизоды ВТЭО и/или получающих терапию антикоагулянтами, отмечены достоверное снижение риска ВТЭО при назначении трансдермального эстрадиола в монорежиме, а также отсутствие увеличения риска ВТЭО при комбинированном использовании трансдермального эстрадиола с гестагеном и перорального эстрадиола с дидрогестероном [34].

По данным наблюдательных исследований, на фоне применения трансдермального эстрадиола в низких (<50 мкг/сут) и более высоких дозах в монорежиме, а также его сочетания с гестагеном в циклическом или непрерывном режимах риск ВТЭО не увеличивался [34–37]. При этом, с одной стороны, есть свидетельства, что трансдермальный путь поступления эстрогенов ассоциируется с более низким риском ВТЭО, чем его пероральный прием, с другой — есть указание на отсутствие различий [34][35][38–40]. Надлежащие рандомизированные контролируемые или иные клинические исследования по сопоставлению этих подходов пока отсутствуют.

В крупном исследовании реальной клинической практики EURAS-HRT (более 30 000 женщин) был подтвержден долгосрочный профиль безопасности дроспиренон-содержащих препаратов для МГТ в отношении ВТЭО. Риск ВТЭО на фоне МГТ с дроспиреноном был сопоставим, а риск серьезных артериальных тромбоэмболических событий (главным образом острого инфаркта миокарда и ишемического инсульта) был достоверно ниже, чем при приеме другой МГТ (детального сопоставления по составу и особенностям другой МГТ не проводилось) [41].

В целом современная низкодозированная и ультранизкодозированная комбинированная пероральная МГТ с использованием эстрадиола представляется безопасной в отношении ВТЭО и по риску венозных тромбозов сопоставимой с трансдермальной МГТ [34][40]. Однако оценка пользы и риска назначения МГТ, выбор лекарственного препарата, его состава и пути введения должны проводиться индивидуально, с учетом особенностей клинической картины и наличия факторов риска ВТЭО.

По данным анализа крупных баз данных QResearch и CPRD, выполненного с использованием метода «случай-контроль», не было отмечено увеличения риска ВТЭО для тиболона [34].

Локальная терапия эстрадиолом симптомов ГУМС не приводит к увеличению риска венозных тромбозов и может использоваться у всех категорий пациенток [31].

ЛНГ-ВМС, содержащая 52 мг микронизированного левоноргестрела, также может быть использована как компонент МГТ. По данным исследований, применение ЛНГ-ВМС не приводило к повышению риска ВТЭО [41][42].

При принятии решения о возможности и составе МГТ следует учитывать, что риск ВТЭО нельзя рассматривать отдельно от других тромботических рисков. Так что даже в случаях, когда не исключено некоторое повышение риска ВТЭО, этот эффект может нивелироваться снижением частоты артериальных тромбозов и других сердечно-сосудистых осложнений, что в итоге обеспечит нейтральное или положительное воздействие на смертность [32][43][44].

## 5.2 МГТ в различных клинических ситуациях, связанных с тромбозами.

Венозные тромбозы

При остром ТГВ и/или ТЭЛА МГТ противопоказана.

Большинство экспертов рекомендуют отказаться от МГТ и у пациенток с ВТЭО в анамнезе [31][45][46]. Есть свидетельства отсутствия увеличения риска рецидива ВТЭО при трансдермальной МГТ на фоне лечения антикоагулянтами, однако данные о безопасности такого подхода после ВТЭО ограничены [37][39].

При тяжелых менопаузальных симптомах помимо локального применения эстрогенов не исключается возможность использования наименьшей эффективной дозы трансдермального эстрадиола (≤50 мкг/сут) или ультранизкодозированной (0,5 мг эстрадиола) пероральной комбинированной МГТ при соответствующей антикоагулянтной терапии [36][37][45][46]. Также не исключено, что современная МГТ достаточно безопасна после планового прекращения использования антикоагулянтов у отдельных категорий больных с низким риском рецидива венозных тромбозов [37].

Имеющиеся данные не позволяют однозначно судить о риске МГТ при остром тромбозе поверхностных вен (ТПВ) и ТПВ в анамнезе [47]. Решение о возможности применения современной пероральной и трансдермальной МГТ при ТПВ должно приниматься индивидуально, с учетом особенностей клинической ситуации, наличия факторов риска ВТЭО, а также наличия ТПВ в анамнезе как противопоказания к применению в инструкции к конкретному препарату.

В исследованиях по оценке риска ТГВ и/или ТЭЛА после перенесенного ТПВ не проводится разделение между тромбозом неварикозных и тромбозом варикозных поверхностных вен (варикотромбофлебитом). Варикотромбофлебит в первую очередь обусловлен наличием варикозного расширения вен, которое может быть устранено задолго до назначения МГТ.

Варикотромбофлебит в анамнезе следует считать ограничением для назначения МГТ при прямом указании на ТПВ в анамнезе как на противопоказание к применению в инструкции к конкретному препарату для МГТ.

Варикозное расширение вен

Наличие варикозного расширения вен не является противопоказанием к МГТ и не должно влиять на принятие решения о назначении МГТ. На сегодняшний день нет данных, что МГТ увеличивает риск развития тромбоза варикозно измененных вен (варикотромбофлебита). Проведение ультразвукового исследования вен нижних конечностей перед назначением МГТ не требуется.

Тромбофилии

Данных о безопасности МГТ при антифосфолипидном синдроме очень мало [39]. Из-за высокого риска венозных и/или артериальных тромбозов пероральная и трансдермальная МГТ у больных с антифосфолипидным синдромом не рекомендуется. Потенциально ее возможность не исключена у женщин с невысокой активностью заболевания или бессимптомными изменениями отдельных лабораторных показателей, не имеющих дополнительных факторов риска тромбозов [47].

Данные о безопасности МГТ при бессимптомных тромбофилиях ограничены. В некоторых исследованиях установлен повышенный риск развития ВТЭО при пероральной МГТ на фоне ряда тромбофилий (дефицит протеина С, дефицит протеина S, дефицит антитромбина, фактор V Лейден, мутация гена протромбина G20210A, высокий уровень фактора свертывания крови VIII) [48][49]. Однако этого недостаточно для однозначного запрета на проведение пероральной МГТ на фоне бессимптомной тромбофилии, требуются дополнительные исследования данного вопроса.

Решение о возможности и составе МГТ следует принимать индивидуально с учетом сведений о наличии ранее выявленной бессимптомной тромбофилии, тяжести менопаузальных симптомов, наличия дополнительных факторов риска ВТЭО, а также указания определенных тромбофилий в перечне противопоказаний в инструкции к конкретному препарату для МГТ [31][40][50]. Обследование на наличие тромбофилий перед началом МГТ не рекомендуется.

Семейный анамнез тромбозов (венозный или артериальный тромбоз у родственников 1 степени родства в возрасте до 50 лет) указывает на повышенный риск ВТЭО, однако не является основанием для запрета МГТ [17][37][51].

По имеющимся данным, трансдермальная МГТ не увеличивает риск ВТЭО у женщин с бессимптомной тромбофилией, однако свидетельства в пользу ее безопасности в этой клинической ситуации ограничены [37][39][49].

Ограничением для применения конкретного препарата является указание на семейный тромботический анамнез и/или наличие определенных тромбофилий как противопоказание к применению в инструкции.

## 5.3 МГТ при хирургических вмешательствах и госпитализации с острым нехирургическим заболеванием

В настоящее время нет доказательств пользы от отмены МГТ перед хирургическими вмешательствами или при госпитализации по поводу острого нехирургического заболевания (кроме тех, при которых МГТ противопоказана) [52]. При повышенном риске ВТЭО профилактика антикоагулянтами нивелирует потенциальный протромботический эффект гормональных препаратов. При стратификации риска ВТЭО у подобных больных продолжение МГТ рекомендуется рассматривать как дополнительный фактор риска ВТЭО.

## РАЗДЕЛ 6. МГТ У ПАЦИЕНТОК С АТЕРОСКЛЕРОТИЧЕСКИМИ СЕРДЕЧНО-СОСУДИСТЫМИ ЗАБОЛЕВАНИЯМИ

В 1998 г. исследование HERS, первое рандомизированное плацебо-контролируемое исследование гормональной терапии (ГТ) эстрогенами и прогестином для вторичной профилактики ишемической болезни сердца (ИБС) среди женщин в постменопаузе с установленной ИБС, не выявило пользы в отношении развития сердечно-сосудистых осложнений и общей смертности при использовании ГТ. Результаты этого исследования являются аргументом против начала ГТ для вторичной профилактики ИБС [53].

Более поздний метаанализ 19 рандомизированных контролируемых исследований с участием 40 410 женщин в постменопаузе, получавших МГТ (большинство из которых принимали перорально), не выявил значительного увеличения смертности от всех причин, смертности от ССЗ или ИМ на фоне МГТ как в рамках первичной, так и в рамках вторичной профилактики сердечно-сосудистых осложнений.

Анализ подгрупп, основанный на сроках начала МГТ, показал:

В настоящее время старт МГТ не рекомендован женщинам с установленным диагнозом ИБС, включая стенокардию [40], а ИМ является противопоказанием к МГТ.

Манифестация ИБС на фоне приема МГТ, как правило, предполагает ее отмену. Хотя авторы уже упомянутого исследования HERS по его результатам заключают, что, учитывая благоприятную картину ишемических событий после нескольких лет МГТ, женщинам с ИБС, уже получающим это лечение, может быть целесообразно продолжить его [53]. Метаанализ, включивший 5766 пациенток с уже имеющимися ССЗ, показал, что абсолютный риск смерти, ИМ, стенокардии или реваскуляризации у этой категории больных на фоне МГТ был низок (табл. 6.1). Таким образом, у пациенток с развившейся в процессе терапии ИБС, настроенных на продолжение МГТ, вопрос о ее отмене должен быть решен индивидуально совместно кардиологом и гинекологом [33].

**Table:** Таблица 6.1. Риск сердечно-сосудистых осложнений и смерти при гормональной терапии у пациенток в постменопаузе с сердечно-сосудистыми заболеваниями (данные метаанализа рандомизированных контролируемых исследований)

Смерть от всех причин	ОР=1,04 (95% ДИ=0,87–1,24)
Смерть от сердечно-сосудистых заболеваний	ОР=1,00 (95% ДИ=0,78–1,29)
Инфаркт миокарда	ОР=0,98 (95% ДИ=0,81–1,18)
Стенокардия	ОР=0,91 (95% ДИ=0,74–1,12)
Реваскуляризация	ОР=0,98 (95% ДИ=0,63–1,53)
Инсульт	ОР=1,09 (95% ДИ=0,89–1,33)

Пациенткам с инсультом в анамнезе рекомендуется избегать системной МГТ и требуется рассмотреть альтернативное (негормональное) лечение. В исследовании WHI повышенный риск ишемического инсульта был отмечен как в группе комбинированной МГТ (ОР=1,37; 95% ДИ=1,07–1,76), так и в группе монотерапии эстрогенами (ОР=1,35; 95% ДИ=1,07–1,70), независимо от исходного риска пациента [54][55]. В метаанализе 4 исследований, включивших 719 участниц без сердечно-сосудистых заболеваний, риск инсульта повышался (ОР =1,32; 95% ДИ =1,12–1,56) по сравнению с плацебо. В метаанализе исследований, выполненных в рамках вторичной профилактики ССЗ (5172 участницы в 5 исследованиях), была отмечена тенденция к увеличению риска инсульта (табл. 6.1) [33].

Неатеросклеротическая/нетромботическая ИБС чаще встречается у женщин, однако в настоящее время недостаточно данных для стратификации риска применения МГТ по подтипам заболевания. Для женщин 50–59 лет с ИМ в анамнезе без обструктивной болезни коронарных артерий, спонтанной диссекции коронарных артерий, коронарной микрососудистой дисфункции или коронарного вазоспазма требуется индивидуальный подход к назначению МГТ. Рекомендуется избегать системной МГТ при спонтанной диссекции коронарных артерий из-за предполагаемой патофизиологической связи с уровнем женских половых гормонов. Эта рекомендация исходит из того факта, что >90% пациентов со спонтанной диссекцией коронарных артерий — женщины.

При симптомах ГУМС женщинам с сердечно-сосудистыми заболеваниями может применяться локальная терапия эстриолом [4][18][56]. Необходимо обратить внимание, что в инструкциях эстрогенов для локального применения содержатся те же противопоказания, что и у эстрогенов для системной МГТ. Это предупреждение основано не на данных научных исследований, а связано с международными требованиями обязательного указания единых противопоказаний для препарата, независимо от путей его введения [45]. Эстриол при локальном применении имеет минимальную системную абсорбцию и не метаболизируется в более активные формы эстрогенов (эстрадиол и эстрон), а уровни циркулирующего эстриола, эстрадиола и эстрона сохраняются в пределах нормальных значений для постменопаузы [57][58]. Несколько крупных обсервационных исследований подтвердили отсутствие повышенного риска неблагоприятных последствий для здоровья, включая ССЗ, ВТЭО и рак при использовании локальной МГТ эстриолом [59][60].

Ключевые положения

## РАЗДЕЛ 7. МГТ У ПАЦИЕНТОК С ФАКТОРАМИ СЕРДЕЧНО-СОСУДИСТОГО РИСКА

## 7.1 Дислипидемии

Клинические исследования показали, что по сравнению с плацебо или отсутствием лечения МГТ может значительно повысить уровень ЛВП, а также снизить уровень ОХС, ХС-ЛНП и Лп(а) [61–63]. Следует отметить, что Лп(а) является независимым фактором риска ССЗ, и в частности повторного ишемического инсульта [64][65]. Статинотерапия оказывает слабое влияние на уровень этого проатерогенного липопротеида, тогда как МГТ достоверно его снижает [66]. Противоречивые данные имеются в отношении действия МГТ на уровень триглицеридов (ТГ). В части исследований имело место достоверное повышение уровня ТГ [67], а в других работах не было обнаружено существенной разницы в ТГ между двумя группами принимающих плацебо и МГТ [62][68–76].

В целом МГТ рассматривается как терапия, связанная с благоприятными изменениями параметров липидов как при кратковременном, так и при длительном применении у женщин в постменопаузе. Однако есть особенности, связанные с дозами препаратов и способом их доставки.

Показано, что пероральная МГТ увеличивает концентрацию ТГ по сравнению с трансдермальной МГТ [63]. Умеренное, но достоверное повышение уровня ТГ даже на фоне терапии фенофибратом и/или полиненасыщенными жирными кислотами может оказать клинически значимое воздействие как на прогрессирование атеросклероза, так и на развитие панкреатита. Таким образом, для женщин с гипертриглицеридемией более безопасным выбором являются трансдермальная или низкодозированная МГТ или тиболон.

В то же время пероральная МГТ связана с положительным влиянием на уровень ХС-ЛНП, а концентрация именно этого проатерогенного фактора в наибольшей степени влияет на развитие атеросклероза и дестабилизацию атеросклеротических бляшек (АСБ).

Вопрос о том, может ли МГТ в низких дозах оказывать такое же влияние на липидный профиль, как и стандартные дозы МГТ, все еще остается неясным. Одно исследование показало, что низкие дозы МГТ были связаны с более высокими уровнями ОХС и ХС-ЛНП, более низким уровнем ТГ, чем стандартные дозы [77]. Другие исследования показали аналогичное преимущество в отношении ТГ в группе низких доз эстрогенов в составе МГТ, но не выявили существенных различий в уровнях ОХС и ХС-ЛНП между двумя группами (высоких и низких доз).

Кроме того, было обнаружено, что низкие дозы эстрадиола в составе МГТ могут снижать уровень ХС-ЛВП. Эпидемиологически низкий уровень ХС-ЛПВП в плазме был связан с повышенным риском ишемических ССЗ [78]. В совокупности преимущество низких доз МГТ и трансдермального пути введения эстрадиола в отношении липидного профиля, возможно, ограничивается только уровнем ТГ.

Существуют противоречивые данные в отношении влияния тиболона на липидный профиль. Метаанализ, проведенный в 2021 г., показал, что тиболон снижает уровни ОХС, ХС-ЛВП и ТГ. Концентрации ЛНП значительно снижаются, если прием тиболона длится ≥26 нед [79]. В отношении влияния на Лп(а) различий между обычной МГТ и тиболоном не наблюдалось [80].

Имеются данные о повышенном риске ИБС у женщин, получавших комбинированную эстроген-гестагенную терапию, но не у женщин, получавших монотерапию эстрогенами [81]. К сожалению, ни в одном крупномасштабном РКИ липидный профиль не оценивался в зависимости от типа используемого прогестагена. Одно из обсервационных исследований показало, что добавление прогестагенов ослабляет благоприятный эффект эстрогена на липидный профиль [82], а метаанализ, проведенный в 2017 г., показал, что не было существенной разницы в снижении концентрации Лп(a) [80].

Хотя результаты ряда исследований продемонстрировали положительное влияние МГТ на липидный профиль, необходимо подчеркнуть, что МГТ не рекомендуется для терапии дислипидемии и снижения риска сердечно-сосудистых заболеваний [83].

Ключевые положения

## 7.2. Артериальная гипертензия

Специфичные для женщин факторы риска АГ и ССЗ в более позднем возрасте включают время наступления менархе, указания в анамнезе на нарушения менструального цикла и репродуктивной функции, миому матки, синдром поликистозных яичников, эндометриоз, неблагоприятные исходы беременности, преждевременную недостаточность яичников и менопаузу. Повышенный риск в течение репродуктивного периода жизни может способствовать более значительному увеличению риска ССЗ в пери- и постменопаузе [84–88].

При АГ, как и при других заболеваниях, выделяют половые и гендерные различия, которые оказывают влияние на эпидемиологию, патофизиологию и клиническое ведение.

В 2019 г. стандартизированная по возрасту распространенность АГ (САД ≥140 мм рт.ст., и/или ДАД ≥90 мм рт.ст., или прием антигипертензивной терапии) во всем мире составила у женщин 32% [89]. При этом в Восточной Европе распространенность АГ у женщин в возрасте 30–79 лет колебалась между 34 и 46% [89]. Распространенность АГ увеличивается с возрастом [90], но имеет более выраженную тенденцию к снижению до наступления менопаузы у женщин, чем у мужчин того же возраста, с заметным повышением у женщин после наступления менопаузы [14]. После 65 лет распространенность АГ у женщин выше, чем у мужчин [89–91].

Траектории АД в течение жизни у мужчин и женщин объясняются различиями механизмов регуляции АД, сочетанием половых и гендерных факторов [89][90]. У женщин до наступления менопаузы эстрогены способствуют снижению АД в контексте их общего вазопротекторного действия. Защита опосредована различными механизмами, в том числе эндотелиальной вазодилатацией за счет усиления пути выработки оксида азота и ингибирования активности симпатической нервной системы и ренин-ангиотензиновой системы. Более того, эстрогены уменьшают выработку эндотелина, окислительный стресс и воспаление [88]. Прекращение функции яичников в результате естественного старения или медицинских вмешательств связано с повышенным бременем кардиометаболических факторов риска, включая увеличение массы тела, уровней глюкозы и холестерина в плазме крови, АД, что приводит к повышению риска ССЗ [87][88][92][93]. После менопаузы заметное снижение уровня эстрогена частично объясняет, почему уровень АД и риск АГ увеличиваются [88][89]. Также в связи с резким снижением уровня прогестерона (природного антагониста альдостерона) происходит реактивация ренин-ангиотензин-альдостероновой системы (РААС) с такими последствиями, как задержка жидкости, повышение артериального давления (АД) [94].

Выделяют следующие специфичные для женщин патофизиологические характеристики АГ [95]:

В постменопаузе у женщин наблюдается более быстрое (по сравнению с мужчинами такого же возраста) увеличение артериальной жесткости. У женщин пожилого возраста отмечается более высокая ригидность аорты, чем у мужчин, что, по-видимому, способствует развитию изолированной систолической АГ, неконтролируемой АГ, сердечной недостаточности с сохраненной фракцией выброса левого желудочка, аортальному стенозу, что чаще встречается у женщин [96][97].

Установлено, что менопауза удваивает риск развития АГ даже после поправки на возраст и индекс массы тела [98]. Хотя МГТ содержит эстрогены, нет убедительных доказательств того, что АД будет значительно повышаться у женщин в менопаузе с АГ или без нее [99]. Однако после начала МГТ необходимо рекомендовать регулярное измерение АД для подтверждения сохраняющегося нормального АД или контроля уровня АД при антигипертензивной терапии [100][101]. В случае неконтролируемой АГ МГТ следует прекратить. Решение от отмене МГТ целесообразно принимать совместно с кардиологом.

Ключевые положения

## 7.3. Курение

Курение значительно увеличивает опасность артериальных сердечно-сосудистых событий и является фактором риска злокачественных новообразований.

Курение не является фактором риска ВТЭО при МГТ (включая комбинированную пероральную МГТ). Несмотря на то что курение само по себе не является основанием для отказа от МГТ, в том числе комбинированными пероральными препаратами, необходимо соблюдать осторожность при назначении пероральной МГТ курильщицам, информировать их о рисках для здоровья, связанных с курением, и настаивать на прекращении курения [23][102][103].

Ключевые положения

## РАЗДЕЛ 8. МГТ В ОСОБЫХ КЛИНИЧЕСКИХ СИТУАЦИЯХ

## 8.1. Атеросклероз периферических артерий

Среди женщин в возрасте 45–49 лет распространенность атеросклероза периферических артерий составляет 4,89%, в возрасте 50–55 лет — 5,73%, в возрасте 56–60 лет — 6,73%. Менопауза увеличивает риск развития каротидного атеросклероза в 2 раза [104]. Преждевременная и ранняя менопауза связана с увеличением объема и распространенности АСБ [105].

Применение монотерапии эстрогенами у женщин в постменопаузе в течение года снижает риск атеросклероза периферических артерий на 52%, как было показано в наблюдательном исследовании Rotterdam study [106]. У больных ишемической болезнью сердца в РКИ HERS и HERSII комбинированная пероральная МГТ не обеспечила статистически значимого снижения количества событий, связанных с атеросклерозом периферических артерий [53][107]. В одном из наблюдательных исследований было определено, что МГТ независимо от ее выбора снижает риск развития атеросклероза периферических артерий на 20% [108]. В описательном обзоре Davies R.S. и соавт. в качестве механизма положительного влияния МГТ на течение периферического атеросклероза обсуждается снижение уровня циркулирующих ЛПНП, повышение уровня ЛПВП и положительное воздействие на функцию эндотелия [109].

## 8.2. Хроническая сердечная недостаточность

В Российской Федерации, по данным популяционного исследования ЭПОХА-ХСН, распространенность ХСН у женщин в возрасте 50 лет составляет 12,2%, в возрасте 60 лет — 26,2%, преимущественно с сохраненной фракцией выброса левого желудочка (ФВ ЛЖ) [112]. Пятилетняя выживаемость больных с ХСН составляет не более 50% [113].

Ранняя менопауза увеличивает риск развития ХСН на 33%, как было выявлено в метаанализе 3 наблюдательных исследований [114].

В РКИ после 10 лет лечения было выявлено, что женщины, получающие пероральную терапию эстрогенами или комбинированную МГТ, назначенную в первые 7 мес в среднем после менопаузы, имели значительно ниже риск смерти, ХСН, инфаркта миокарда без какого-либо увеличения риска рака, ВТЭО или инсульта [115].

Пероральная терапия эстрогенами и комбинированная МГТ у пациенток 50 лет и старше с ХСН III–IV функционального класса и ФВ ЛЖ ≤35% неишемической этиологии обеспечила статистически значимое снижение риска общей смертности на 40%, как было продемонстрировано в субанализе РКИ BEST (Beta-Blocker Evaluation of Survival Trial) [116].

Субанализ РКИ WHI (Women’s Health Initiative) показал, что монотерапия пероральными эстрогенами и комбинированная МГТ не увеличивает риск госпитализаций, связанных с ХСН, независимо от ФВ ЛЖ и возраста женщины при назначении МГТ [117].

## 8.3. Фибрилляция предсердий

Известно, что женщины во всех возрастных группах имеют более низкую распространенность фибрилляции предсердий (ФП) по сравнению с мужчинами, но смертность от всех причин у женщин выше: ФП независимо связана с 2-кратным увеличением риска смерти у женщин по сравнению с 1,5-кратным увеличением риска смерти у мужчин [118]. В наблюдательном исследовании ATRIA ежегодная частота тромбоэмболических осложнений у пациентов с ФП, не принимающих варфарин, составила 3,5% для женщин по сравнению с 1,8% для мужчин [119]. Женщины с дополнительными факторами риска инсульта, особенно в старшем возрасте (>65 лет), подвергаются большему риску инсульта, даже если они принимают антикоагулянтную терапию, в то время как риск кровотечения при антикоагуляции был одинаков у обоих полов [120]. У женщины с ФП более выражена симптоматика и более тяжелые инсульты. В клинической практике женщины с ФП реже получают специализированную помощь, чаще применяется более консервативный подход [121][122].

Наличие менопаузы увеличивает риск ФП на 82% [123].

Данные наблюдательного исследования BiomarCaRE Consortium в Европе продемонстрировали, что у женщин в постменопаузе (средний возраст 49,2 года) распространенность ФП составила 4,4%, что было взаимосвязано с увеличением риска инсульта на 42%, инфаркта миокарда на 78%, а частота смертельных исходов возрастала более, чем в 3,5 раза [124].

По данным субанализа РКИ WHI и наблюдательных исследований комбинированная МГТ, монотерапия пероральными эстрогенами, применение тиболона увеличивают риск развития ФП [123][125–127].

Вклад трансдермальных и локальных форм эстрогенов в развитие ФП у женщин в период менопаузы не определен.

## 8.4. Патология клапанов сердца

Возможность назначения пероральной МГТ у женщин в пери- и постменопаузе с патологией клапанов определяется наличием осложнений:

## ЗАКЛЮЧЕНИЕ

Показания и противопоказания к назначению МГТ определяются актуальными Клиническими рекомендациями и инструкциями к конкретным препаратам.

## ДОПОЛНИТЕЛЬНАЯ ИНФОРМАЦИЯ

Источники финансирования. Работа выполнена по инициативе авторов без привлечения финансирования

Конфликт интересов. Авторы декларируют отсутствие явных и потенциальных конфликтов интересов, связанных с содержанием настоящей статьи.

Участие авторов. Все авторы одобрили финальную версию статьи перед публикацией, выразили согласие нести ответственность за все аспекты работы, подразумевающую надлежащее изучение и решение вопросов, связанных с точностью или добросовестностью любой части работы.

## ПРИЛОЖЕНИЕ 1.

**Table:** Таблица. Критерии приемлемости назначения МГТ Категория 1 — отсутствие ограничений на использование МГТ;Категория 2 — преимущества перевешивают риски;Категория 3 — риски, как правило, перевешивают преимущества;Категория 4 — МГТ не должна использоваться.НП — неприменимо из-за отсутствия данных.

	Комбинированная МГТ	Монотерапия эстрогенами	Тиболон	Локальная МГТ	Примечания
Перорально	Трансдермально	Перорально	Трансдермально
Нарушения углеводного обмена
СД	1	1	2	1	НП	1	
Венозные тромбозы и/или ТЭЛА
Острый ТГВ/ТЭЛА	4	4	4	4	4	1	Под острыми ТГВ/ТЭЛА понимается период, требующий использования полной лечебной дозы антикоагулянта (основная фаза антикоагулянтной терапии, первые 3–6 мес).
ТГВ/ТЭЛА в анамнезе	4	3	4	3	4	1	При тяжелых менопаузальных симптомах во время лечения антикоагулянтами у отдельных больных можно рассмотреть трансдермальную или ультранизкодозированную пероральную МГТ; в большинстве случаев МГТ не следует использовать после отмены антикоагулянтов.
Тромбоз поверхностных вен (острый или в анамнезе)	3	3	3	3	НП	1	
Нетромботические хронические заболевания вен
Нетромботические хронические заболевания вен (варикозное расширение вен, ретикулярные вены, телангиэктазы нижних конечностей)	1	1	1	1	1	1	
Тромбофилии
Бессимптомная тромбофилия с высоким риском ВТЭО (дефицит протеина S, дефицит протеина С, дефицит антитромбина, фактор V Лейден, мутация гена протромбинa G20210A, высокий уровень фактора свертывания крови VIII)	3	2	3	2	НП	1	Необходимо учитывать ранее выявленную тромбофилию, рутинное обследование на тромбофилию перед назначением МГТ не требуется. Решение о возможности и составе МГТ следует принимать индивидуально на основании сведений о наличии ранее выявленной бессимптомной тромбофилии, тяжести менопаузальных симптомов, дополнительных факторов риска ВТЭО, а также указания определенных тромбофилий в перечне противопоказаний в инструкции к конкретному препарату для МГТ. По имеющимся данным, трансдермальные препараты для МГТ не повышают риск венозных тромбозов у пациенток с бессимптомной тромбофилией.
Антифосфолипидный синдром	4	3	4	3	4	1	Возможность МГТ не исключена у женщин с низкой или умеренной активностью заболевания, не имеющих дополнительных факторов риска венозных тромбозов.
Семейный анамнез тромбозов	2	2	2	2	2	1	Наличие родственника 1 степени родства, перенесшего венозный или антериальный тромбоз в возрасте до 50 лет.
Хирургические вмешательства и острые нехирургические заболевания с госпитализацией
Хирургическое вмешательство	1	1	1	1	1	1	Перед проведением хирургического вмешательства необходима оценка риска развития ТГВ/ТЭЛА в послеоперационном периоде по шкале Каприни. Рекомендуется при оценке риска развития послеоперационных ТГВ/ТЭЛА учитывать проведение МГТ как 1 дополнительный балл по шкале Каприни. Отмена МГТ при хирургических вмешательствах не требуется. Профилактика венозных тромбозов антикоагулянтами должна проводиться в соответствии с определенной по шкале Каприни категорией риска развития ТГВ/ТЭЛА.
Острые нехирургические заболевания, требующие госпитализации	1	1	1	1	1	1	При госпитализации необходима оценка риска развития ТГВ/ТЭЛА по рекомендуемым шкалам (например, шкала Padua). Рекомендуется при оценке риска развития послеоперационных ТГВ/ТЭЛА учитывать проведение МГТ как 1 дополнительный балл. Отмена МГТ при острых нехирургических заболеваниях, требующих госпитализации, не входящих в состав противопоказаний к МГТ, не требуется. Профилактика венозных тромбозов антикоагулянтами должна проводиться в соответствии с определенной по шкале категорией риска развития ТГВ/ТЭЛА.
Атеросклеротические сердечно-сосудистые заболевания
ИБС	3	3	3	3	НП	1	При наличии ИБС старт МГТ не рекомендован. У пациенток с развившейся в процессе терапии ИБС, настроенных на продолжение МГТ, вопрос о ее отмене должен быть решен индивидуально кардиологом и гинекологом совместно.
Инфаркт миокарда (острый или в анамнезе)	4	4	4	4	4	1	
Нарушение мозгового кровообращения, включая транзиторную ишемическую атаку (острое или в анамнезе)	4	4	4	4	4	1	
Факторы риска ССЗ
Гиперлипидемия (кроме гипертриглицеридемии)	1	1	1	1	1	1	
Гипертриглицеридемия	3	2	3	2	2	1	При уровне ТГ>4,5 ммоль/л не рекомендован старт МГТ, требуется коррекция уровня ТГ.
Артериальная гипертония	1	1	1	1	1	1	МГТ может быть назначена при условии контроля АД.
Курение	2	2	2	2	НП	1	У курящих особое внимание необходимо уделять совокупности факторов риска и с их учетом решение принимать индивидуально.
Другие заболевания/состояния
Атеросклероз периферических артерий	2	2	2	НП	НП	НП	
Хроническая сердечная недостаточность (неишемического генеза)	2	2	2	НП	НП	НП	
Фибрилляция предсердий	4	4	4	4	4	НП	

## ПРИЛОЖЕНИЕ 2.

**Figure:**
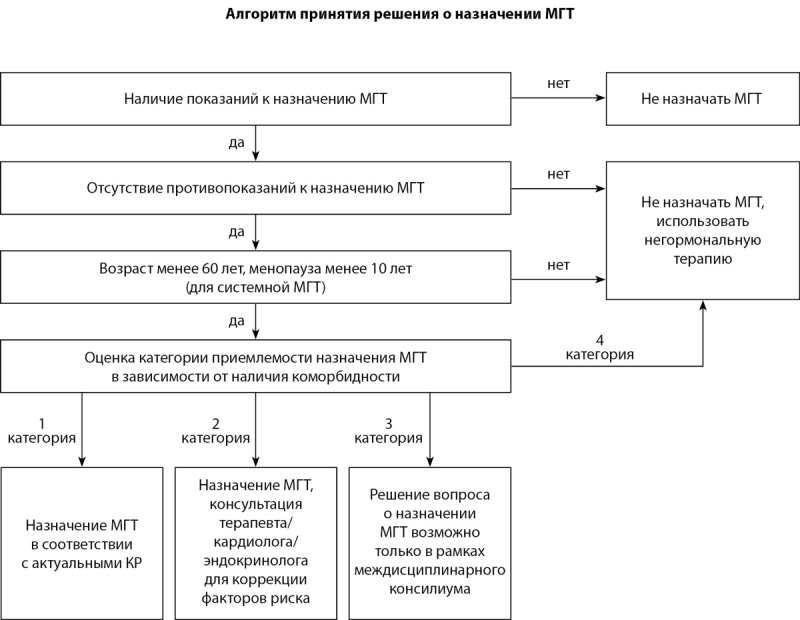


## ПРИЛОЖЕНИЕ 3.

**Figure:**